# Mechanisms of repeat-associated non-AUG translation in neurological microsatellite expansion disorders

**DOI:** 10.1042/BST20200690

**Published:** 2021-03-17

**Authors:** Lydia M. Castelli, Wan-Ping Huang, Ya-Hui Lin, Kung-Yao Chang, Guillaume M. Hautbergue

**Affiliations:** 1 Sheffield Institute for Translational Neuroscience (SITraN), Department of Neuroscience, University of Sheffield, Sheffield S10 2HQ, U.K.; 2 Institute of Biochemistry, Life Science Building, National Chung-Hsing University, Taichung City 402, Taiwan; 3 Neuroscience Institute, University of Sheffield, Western Bank, Sheffield S10 2TN, U.K.

**Keywords:** microsatellite repeat expansion disorders, pathophysiology, RAN translation

## Abstract

Repeat-associated non-AUG (RAN) translation was discovered in 2011 in spinocerebellar ataxia type 8 (SCA8) and myotonic dystrophy type 1 (DM1). This non-canonical form of translation occurs in all reading frames from both coding and non-coding regions of sense and antisense transcripts carrying expansions of trinucleotide to hexanucleotide repeat sequences. RAN translation has since been reported in 7 of the 53 known microsatellite expansion disorders which mainly present with neurodegenerative features. RAN translation leads to the biosynthesis of low-complexity polymeric repeat proteins with aggregating and cytotoxic properties. However, the molecular mechanisms and protein factors involved in assembling functional ribosomes in absence of canonical AUG start codons remain poorly characterised while secondary repeat RNA structures play key roles in initiating RAN translation. Here, we briefly review the repeat expansion disorders, their complex pathogenesis and the mechanisms of physiological translation initiation together with the known factors involved in RAN translation. Finally, we discuss research challenges surrounding the understanding of pathogenesis and future directions that may provide opportunities for the development of novel therapeutic approaches for this group of incurable neurodegenerative diseases.

## Introduction

Microsatellite expansions have been characterised in a large number of incurable neurodegenerative diseases subdivided into polyglutamine (polyQ) and non-polyglutamine (non-polyQ) disorders [1]. Autosomal-dominant glutamine-encoding CAG repeat expansions in the Huntingtin gene (*HTT*) cause Huntington's disease (HD) [2,3] while CAG repeats in the coding regions of various unrelated ataxin genes lead to spinocerebellar ataxias (SCA) [4,5]. Non-polyQ expansion disorders are caused by various lengths of trinucleotide to hexanucleotide repeat sequences mostly contained within non-coding regions of genes (5′-/3′-untranslated regions (UTR) and introns). These most commonly include: CGG repeats in fragile X mental retardation 1 (*FMR1*) gene in Fragile X-associated syndromes [6]; thousands of CTG/CCTG repeats in the myotonic dystrophies (DM1 and DM2) [7,8] and trinucleotide, pentanucleotide or hexanucleotide repeats in non-polyQ SCAs [9]; GAA repeat expansions in Friedreich's ataxia [10]; thousands of GGGGCC repeats in chromosome 9 open reading frame 72 (*C9ORF72*) in the most common genetic forms of amyotrophic lateral sclerosis (ALS) and frontotemporal dementia (FTD) [11,12]. Altogether, we compiled a list of 53 expansion disorders that mainly present with neurodegenerative conditions (Table 1).

**Table 1:** Microsatellite repeat expansion disorders

Disorder	Gene	Sense repeat	Antisense repeat	Disease length	Location in gene	RAN translated proteins	References
**PolyQ microsatellite repeat expansion disorders:**							
Dentatorubropallidoluysian Atrophy (DRPLA)	*ATN1/DRPLA*	CAG	Unknown	49–88	Exon 5	Unknown	[127]
Schizophrenia/migraines	*KCNN3*	CAG	Unknown	>28	Exon 1	Unknown	[128]
Prostate/breast Cancer	*AIB/SRC-3*	CAG/CAA	Unknown	>23	Exon 20	Unknown	[129]
Huntington's Disease (HD)	*HTT*	CAG	CTG	36–250	Exon 1	polyS, polyA, polyC, polyL in human brains & *in vitro*^3^	[2,52]
Spinal and Bulbar Muscular Atrophy (SBMA)	*AR*	CAG	Unknown	38–62	Exon 1	Unknown	[130]
Spinocerebellar Ataxia Type 1 (SCA1)	*ATXN1*	CAG	Unknown	49–88	Exon 8	Unknown	[131]
Spinocerebellar Ataxia Type 2 (SCA2)	*ATXN2*	CAG	CTG	33–77	Exon 1	polyQ, polyA, polyS *in vitro*^3^	[132,133]
Spinocerebellar Ataxia Type 3 (SCA3) or Machado-Joseph Disease (MJD)	*ATXN3*/*MJD*	CAG	CTG	55–86	Exon 10	polyQ, polyA, polyS *in vitro*^3^	[45,108,134]
Spinocerebellar Ataxia Type 6 (SCA6)	*CACNA1A*	CAG	Unknown	21–30	Exon 47	Unknown	[135]
Spinocerebellar Ataxia Type 7 (SCA7)	*ATXN7*	CAG	Unknown	28–120	Exon 3	Unknown	[136]
Spinocerebellar Ataxia Type 17 (SCA17)	*TBP*	CAG/CAA	Unknown	47–63	Exon 3	Unknown	[137]
**Non-polyQ microsatellite repeat expansion disorders:**							
Amyotrophic lateral sclerosis (ALS)/Frontotemporal Dementia (FTD)	*C9ORF72*	GGGGCC	CCCCGG	30–4400	Intron 1	polyGA, polyGP, polyGR, polyPA, polyPR in human brains & *in vitro*^3^	[11,12,49–51,120]
Baratela-Scott Syndrome	*XYLT1*	GGC	Untranscribed expansion	>100	Promoter	Unknown	[138]
Blepharophimosis-Ptosis-Epicanthus Inversus Syndactylyl	*FOXL2*	GCG	Unknown	22–24	Exon 1	Unknown	[139]
Cerebellar Ataxia, Neuropathy, Vestibular Areflexia Syndrome (CANVAS)	*RFC1*	AAGGG	Unknown	400–2000	Intron 2	Unknown	[140]
Cleidocranial Dysplasia	*RUNX2*/*CBFA1*	GCG	Unknown	>20	Exon 1	Unknown	[141]
Congenital Central Hypoventilation/Haddad Syndrome	*PHOX2B*	GCG	Unknown	5–13	Exon 3	Unknown	[142]
Familial adult myoclonic epilepsy (FAME1/BAFME1)	*SAMD12*	TTTCA/TTTTA	Unknown	440–3680	Intron 4	Unknown	[143]
Fragile X syndrome (FRAXA/FXS)	*FMR1*	CGG	CCG	>230	5′-UTR	Unknown	[6]
Fragile X-associated tremor/ataxia syndrome (FXTAS)	*FMR1*	CGG	CCG	55–200	5′-UTR	polyG, polyP, polyA, polyR *in vitro*^3^ and *Drosophila*	[46,144,145]
Fragile X-associated Primary Ovary Insufficiency (FXPOI)	*FMR1*	CGG	Not found^1^	55–200	5′-UTR	polyG in biopsied human ovarian stromal cells	[146,147]
Fragile XE mental retardation (FRAXE)	*AFF2*/*FMR2*	CGG/CCG	Untranscribed expansion	>200	Promoter	Unknown	[17]
Fragile XF syndrome (FRAXF)	*TMEN185A*	GCC	Unknown	300–500	5′-UTR	Unknown	[148]
FRA2A-associated mental retardation	*AFF3*	CGG	Unknown	>300	5′-UTR	Unknown	[19]
FRA7A-associated autism spectrum disorder	*ZNF713*	CGG	Unknown	>85	Intron 1	Unknown	[19]
FRA10A-associated mental retardation	*FRA10AC1*	CGG	Unknown	>200	5′-UTR	Unknown	[20]
FRA11A-associated mental retardation	*C11ORF80*	CGG	Unknown	500	5′UTR	Unknown	[149]
FRA12A-associated mental retardation	*DIP2B*	CGG	Unknown	>50	5′-UTR	Unknown	[21]
FRA16A-associated mental retardation	*LOC109617027*	CGG	Unknown	1000–1900	5′-UTR	Unknown	[150]
Friedreich's ataxia (FRDA)	*FXN*/*X25*	GAA	TTC	>100	Intron 1	Unknown	[10]
Fuchs’ Endothelial Corneal Dystrophy (FECD)	*TCF4*	CTG	CAG	>50	Intron 3	polyC in human corneal endothelium + polyA, polyQ, polyS *in vitro*^3^	[151,152]
Hand-Foot-Genital Syndrome	*HOXA13*	GCG	Unknown	24–26	Exon 1	Unknown	[153]
Holoprosencephaly	*ZIC2*	GCG	Unknown	>25	Exon 3	Unknown	[154]
Huntington Disease-Like 2 (HDL2)	*JPH3*	CAG	CTG	>41	3′-terminal exon	polyQ, polyA, polyS *in vitro*^3^	[45,155]
Jacobsen Syndrome	*FRA11B*/*CBL2*	CGG	Not found^1^	100–1000	5′-UTR	Unknown	[156]
Myoclonus Epilepsy of the Unverricht-Lundborg Type	*CYSTB*	CCCCGCCCCGCG	Untranscribed expansion	12–13	Promoter	Unknown	[18]
Congenital Myotonic Dystrophy (CDM)/Steinert's Disease	*DMPK*	CTG	CAG	50–10000	3′-UTR	Unknown	[157]
Myotonic dystrophy (DM1)	*DMPK*	CTG	CAG	50–10000	3′-UTR	polyQ in human muscle/blood + polyA, polyS *in vitro*^3^	[7,8,45,157]
Myotonic dystrophy type 2 (DM2)	*ZNF9*	CCTG	GGAC	75–1100	Intron 1	polyQAGRpolyLPAC in human brains & *in vitro*^3^	[106,158]
Neuronal Intranuclear Inclusion Disease (NIID) & Amyotrophic lateral Sclerosis (ALS)	*NOTCH2NLC*	GGC	Unknown	>71	5′-UTR	Unknown	[159,160]
Oculopharyngeal Musclar Dystrophy	*PABPN1/PABP2*	GCG	Unknown	12–17	Exon 1	Unknown	[161]
Pseudoachrondroplasia and Multple Epiphyseal Displaysia (PSACH/MED)	*COMP*	GAC	Not found^1^	>6	Exon 13	Unknown	[162]
Spinocerebellar Ataxia Type 8 (SCA8)	*ATXN8OS & ATXN8*	CTG	CAG	110–250	3′-UTR *ATXN8OS*; 5′UTR *ATXN8*	polyA in human brains + polyS, polyQ *in vitro*^3^	[15,45]
Spinocerebellar ataxia Type 10 (SCA10)	*ATXN10*	ATTCT	Not found^1^	32–4000	Intron 9	Unknown	[163]
Spinocerebellar ataxia Type 12 (SCA12)	*PPP2R2B*	CAG	CTG	66–78	5′-UTR	Unknown	[164]
Spinocerebellar ataxia Type 31 (SCA31)	*BEAN1*	TGGAA	TTCCA	>110	Intron 1	polyWNGME?^2^ *in vitro*^3^	[165]
Spinocerebellar ataxia Type 36 (SCA36)	*NOP56*	GGCCTG	Unknown	>100	Intron 1	polyGP, polyPR in human brains + polyGL, polyWA *in vitro*^3^	[166,167]
Spinocerebellar ataxia Type 37 (SCA37)^4^	*DAB1*	ATTTC	GAAAT	31–75	5′-UTR	Unknown	[168]
Synpolydactylyl Type II (SPD)	*HOXD13*	GCG	Unknown	22–29	Exon 1	Unknown	[169]
X-Linked Dystonia-Parkinsonism (XPD)^5^	*TAF1*	CCCTCT	Unknown	35–52	Intron 32	Unknown	[170]
X-Linked Mental Retardation and Abnormal Genitalia (XLAG)	*ARX*	GCN	Unknown	20	Exon 2	Unknown	[171]
X-Linked Mental Retardation (XMLR)	*ARX*	GCN	Unknown	18–23	Exon 2	Unknown	[172]
X-linked Mental Retardation with Growth Hormone Deficiency (XLMRGHD)	*SOX3*	GCN	Unknown	15–26	Exon 1	Unknown	[173]

1 Not found indicates that antisense transcripts were not detected;

2 The polypeptide polyWNGME is produced from the intronic repeat expansion, however it can not be confirmed as a RAN translation product due to the presence of an ATG sequence encoding a canonical AUG start codon within the repeat expansion;

3 *In vitro* indicates that the RAN-translated proteins were detected from reporter constructs in transfected cell model of diseases;

4 Not classical expansions but insertions due to replication/recombination/duplication events;

5 Not classical expansion but insertion due to retrotransposon event.

## Pathogenic mechanisms induced by microsatellite repeat expansions

The transcription of repeat expansions located in coding and non-coding regions of genes generates pathological transcripts with polymorphic RNA-repeat sequences. The microsatellite loci are moreover bi-directionally transcribed in HD, DM, C9ORF72-ALS/FTD and in some SCAs and Fragile X-associated syndromes leading to expression of both sense and antisense repeat transcripts. These are thought to cause neuronal injury through complex intertwined mechanisms involving: (i) translation of proteins with expanded stretches of glutamine in poly-Q disorders; (ii) protein gain-of-functions caused by repeat-associated non-AUG (RAN) translation of toxic repeat proteins; (iii) RNA toxic gain-of-functions through the sequestration of RNA-binding proteins within RNA foci and onto repeat transcripts; (iv) protein loss-of-functions via haploinsufficiency (reviewed in [13,14]).

### Translation of protein with expanded poly-glutamine domains

Polymorphic CAG repeat expansions in HD and poly-Q SCAs encode long stretches of poly-glutamine and the translation of proteins with polyQ domains. These promote misfolding/ aggregation, inhibit interactions with physiological binding protein partners and generate abnormal interactions with other proteins, mediating thus both toxic protein loss- and gain-of-functions [4]. The non-polyQ disorder SCA8 was initially shown to express expanded CUG repeats in the 3′-UTR of the *ATXN8OS* (*ATXN8 Opposite Strand*) gene [15]. Later, bidirectional expression of CAG expansion transcripts from *ATXN8* were reported and shown to result in the expression and accumulation of a polyQ protein that forms neuronal inclusions [9].

### Haploinsufficiency

Loss-of-function of the genes harbouring the repeat expansions can directly contribute to the pathophysiology of the microsatellite repeats. Over 200 CGG repeats in the 5′-UTR of *FMR1* cause Fragile X syndrome (FXS) [16], the most common inherited form of intellectual disability, due to transcriptional silencing induced by DNA methylation of the CGG trinucleotides and loss of the FMRP protein which has roles in synaptic plasticity. A contributory loss-of-function is the likely pathological cause of diseases where the repeat expansions are found in promotors, e.g. fragile-XE mental retardation (*FMR2* gene; [17]) and myoclonus epilepsy of the Unverricht-Lundborg type (*CYSTB* gene; [18]). Loss-of-function is also associated with folate sensitive fragile sites harbouring CGG repeats (FRA7A, FRA10A and FRA12A) through DNA methylation of the repeat expansions [19–21]. Hexanucleotide-repeat expansions in the 5′-UTR region of *C9ORF72* lead to decreased expression levels of *C9ORF72* mRNAs, encoding a protein involved in autophagy regulation [22–25], vesicle trafficking [26,27] and immune response in mice [28,29] in several *in vitro* and *in vivo* models and post-mortem brains [11,30–33]. However, the direct contribution of reduced levels of C9ORF72 protein to disease pathogenesis is still debated.

### Formation of RNA foci and RNA-repeat sequestration of proteins

RNA-mediated cellular toxicity results in either protein gain- and loss- of-functions via sequestration of RNA-processing proteins on repeat transcripts which may either be co-transcriptionally processed or aggregated into RNA foci. Protein loss-of-functions have been implicated in a wide range of expansion disorders via RNA-repeat sequestration of mRNA-binding proteins which may loose their normal cellular functions including: muscleblind-like splicing regulator (MBLN) and CUG-binding protein and ETR3-like factor (CELF) families of proteins in myotonic dystrophy [34–36]; MBLN and other RNA-binding proteins in polyQ disorders [37,38]; Sam68 [39], PUR-alpha, hnRNP A2/B1, CUGBP1 [40,41] in fragile X-associated tremor ataxia syndrome (FXTAS); PUR-alpha, heterogeneous nuclear ribonucleoproteins (hnRNPs) and SR-rich splicing factors (SRSFs) among others in C9ORF72-ALS/FTD [42,43]. On the other hand, toxic protein gain-of-function also occurs through RNA-repeat sequestration of SRSF1 which triggers the nuclear export and subsequent RAN translation of sense and antisense *C9ORF72*-repeat transcripts retaining pathological expansions in intron-1 [44].

### RAN translation of toxic repeat proteins

In 2011, Laura Ranum's group demonstrated that CAG-repeat transcripts lacking canonical AUG start codons are remarkably translated into homo-polymeric proteins in all frames (poly-glutamine, poly-serine and poly-alanine) by repeat-associated non-AUG (RAN) translation [45]. RAN-translated poly-alanine proteins driven from *ATXN8* CAG-repeat transcripts were also characterised in SCA8 mice and human brain tissue [45]. Interestingly, the poly-alanine repeat proteins can also be produced by RAN translation of the 5′UTR-sense *ATXN8OS* CUG-repeat transcripts in transfected cells. Since this discovery, RAN translation of non-coding transcript regions was highlighted to occur from CGG repeats in FXTAS which produce toxic poly-glycine FMRpolyG and poly-alanine FMRpolyA proteins [46–48] and from bi-directionally transcribed GGGGCC repeats in all frames in C9ORF72-ALS/FTD to generate five cytotoxic sense and antisense dipeptide-repeat proteins (DPRs) (poly-glycine-alanine, poly-glycine-arginine, poly-glycine-proline, poly-proline-alanine and poly-proline-arginine) [49–51]. Moreover, RAN translation also occurs through the coding CAG-repeat expansions in the *HTT* open reading frame leading overall to both canonical translation of the polyQ-expanded HTT mutant protein and to four RAN-translated sense and antisense homo-polymeric repeat proteins in HD (poly-alanine, poly-serine, poly-leucine, poly-cysteine) [52]. To date, RAN translation has been evidenced from repeat transcripts expressed in human disease samples in seven expansion disorders (Table 1).

The recent discovery of RAN translation challenged the initial hypothesis that non-coding repeat expansion disorders are primarily caused by RNA foci and protein loss-of functions due to sequestration of RNA-binding proteins since polymeric repeat proteins exhibit aggregating properties and high levels of cytotoxicity in multiple cell and animal models of repeat expansion disorders. A range of polypeptides are produced through these mechanisms from the homo-polymeric proteins derived from trinucleotide repeat expansions through to dipeptide repeat proteins found in C9ORF72-ALS/FTD and SCA36 to more complex polypeptide repeat proteins expressed in transfected reporter cell models in SCA31 and DM2 (Table 1). Repeat expansions can be translated from sense, e.g. Jacobsen Syndrome, or sense and antisense strands, e.g. C9ORF72-ALS/FTD, SCA8, HD and FXTAS. The pathophysiological properties of C9ORF72-ALS/FTD DPRs are the most characterised. Increasing evidence has associated very high cytotoxicity to the arginine-containing DPRs (poly-glycine-arginine and poly-proline-arginine) in *Drosophila*, mice, patient-derived neurons and other cell models [53–59] while poly-GA toxicity was also reported in chicks and mice [60–62]. Mechanisms of DPR-mediated cytoxicity include nucleolar dysfunction [53], transcriptional silencing [63], broad disruption of gene expression through interaction with low complexity domain-containing proteins such as RNA Recognition Motif proteins [64], altered splicing [65] and nucleocytoplasmic transport [44,66,67], impairment of DNA repair [68], mitochondrial defects [59,69] and global alteration of translation [56,70,71] together with alterations of ubiquitin/proteasome mediated proteolysis [72,73].

## Physiological mechanisms of eukaryotic translation

Translation involves three distinct mechanisms in Eukaryotes: (i) canonical AUG-driven cap-dependent initiation of translation for the vast majority of mRNAs; (ii) IRES-mediated cap-independent translation and (iii) canonical translation using alternative near-cognate codons.

**Figure 1.**
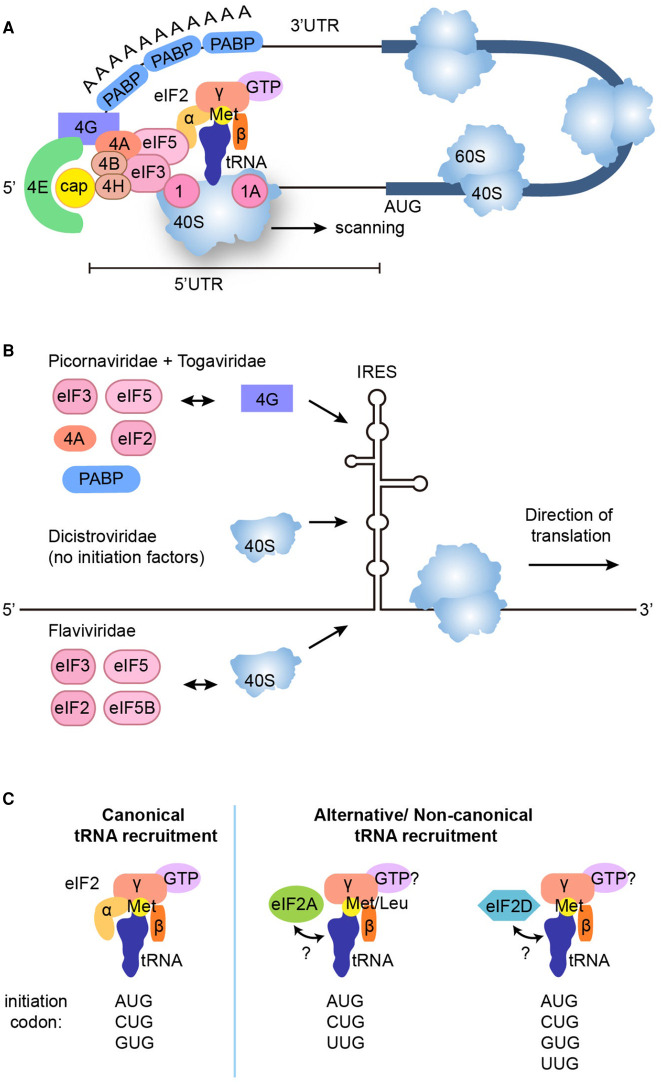
Canonical and physiological translation initiation mechanisms. (**A**) Canonical initiation involves the eIF4F complex and the poly(A) tail binding protein PABP binding to the mRNA and subsequently interacting with the 43S complex (eIF5, eIF3, eIF2 and the 40S ribosome) to form the 48S complex. eIF4E and PABP both interact with eIF4G to create a ‘closed loop complex'. eIF4A, with its cofactors eIF4B and eIF4H, interact with eIF4G and eIF4E to provide helicase activity to unwind secondary structures present in the 5′UTR. The 48S complex scans the mRNA for an AUG start codon, where the 60S ribosomal subunit is recruited through eIF5B and several of the initiator factors are displaced and recycled to initiate a new round of translation. (**B**) IRES mediated translation involves a strong secondary or tertiary structure within the 5′UTR. The precise mechanisms vary between viruses but the IRES element interacts with either the 40S subunit or eIF4G, which recruit any other required factors to initiate translation. (**C**) Canonical and alternative physiological initiator tRNA-binding eIF factors recognise different start codons. Canonical translation occurs through eIF2α, delivering Met-tRNA_i_^Met^ to the P site of the 40S ribosomal subunit in a GTP-dependent manner, through interaction with both the canonical AUG start codon and near cognate start codons CUG and GCG. Both eIF2A and eIF2D are also able to initiate translation, however this can occur in a GTP-dependent or independent manner, binding either charged or uncharged tRNA_i_^Met^. eIF2A can additionally bind Leu-tRNA^CUG^ to initiate translation. eIF2A can initiate translation at AUG, CUG and UUG codons, while eIF2D can initiate at AUG, CUG, GCG and UUG codons.

Translation initiation of canonical mRNAs is a complex process which requires many eukaryotic initiation factors (eIFs) and is one of the key rate-limiting steps for the regulation of gene expression [74]. Translation of canonical mRNAs has been shown to occur through the formation of a closed loop complex, with eIF4G forming a bridge between the m7cap-binding protein eIF4E and the poly(A) tail binding protein PABP although the closed loop formation does not explain initiation of all cellular mRNAs [75]. Briefly, the 40S ribosomal subunit is recruited to mRNAs upstream of the translation start site via multiple initiation factors and an incorporated eIF2α-bound Met-tRNAi^Met^ to form the 48S pre-initiation complex, which scans along the mRNA 5′-UTR with the RNA-helicase eIF4A and its cofactors eIF4B and eIF4H unwinding any secondary structures until the AUG codon is reached. Further initiation proteins facilitate the joining of the 60S subunit to produce the initiating 80S complex [74]. Regulation of translation is predominantly exerted at the initiation stage where the AUG start codon is identified by eIF2α-bound methionyl-tRNA and start codon selection efficiency is tightly influenced by the surrounding nucleotide sequence known as the Kozak consensus element [76]. A schematic of canonical AUG-driven translation initiation is provided in Figure 1A.

Alternative initiation mechanisms using internal ribosome entry site (IRES) elements are utilised by many viral and a growing number of cellular mRNAs [77]. IRES elements drive translation in a cap-independent manner via distinct secondary or tertiary RNA structures that directly bind either the initiation factor eIF4G (picornaviridae and togaviridae) or the 40S ribosome (dicistroviridae and flaviviridae) to initiate translation [78,79] (Figure 1B). With picornaviridae, togaviridae and flaviviridae, eIF4G or the 40S ribosome subsequently recruits other initiation factors to facilitate translation of the IRES-harbouring RNAs while IRES from dicistroviridae require no initiation factors directing translation solely via its binding of the 40S ribosome (Figure 1B).

Finally, near-cognate start codons (typically CUG, GUG and UUG), which differ from the AUG start codon by one nucleotide, initiate translation in mammalian cells at a much lower efficiency, using the non-AUG initiator tRNAi^Met^ and methionine as the initiating amino acid [80] or an elongator Leu-tRNA^Leu^ at a CUG codon in the case of the major histocompatibility complex class I molecules [81,82]. Near-cognate initiation sites can be used by mismatch recognition of eIF2α-bound Met-tRNAi^Met^ [80], when fidelity of start codon usage is affected depending on secondary structures downstream of the initiation codon and expression levels of other initiation factors such as eIF1 or eIF5 which respectively increases or decreases the fidelity of AUG recognition. Two other initiation factors (eIF2A and eIF2D) can also be used at non-AUG codons to initiate translation in either a GTP-dependent manner through initiator tRNAi^Met^ or a GTP-independent manner through Leu-tRNA^Leu^ [83] (Figure 1C).

## Mechanisms of RAN translation

RAN translation involves the translation of short repeated RNA sequences in sense and/or antisense transcripts in an AUG-independent manner and in multiple frames. However, how RAN translation occurs and which sets of factors are required for initiation, elongation and potential regulatory controls remains largely unknown, although it is clearly emerging that some features are shared with canonical and/or IRES-mediated initiation [84].

### The roles of initiation factors and RNA structures in RAN translation

**Figure 2.**
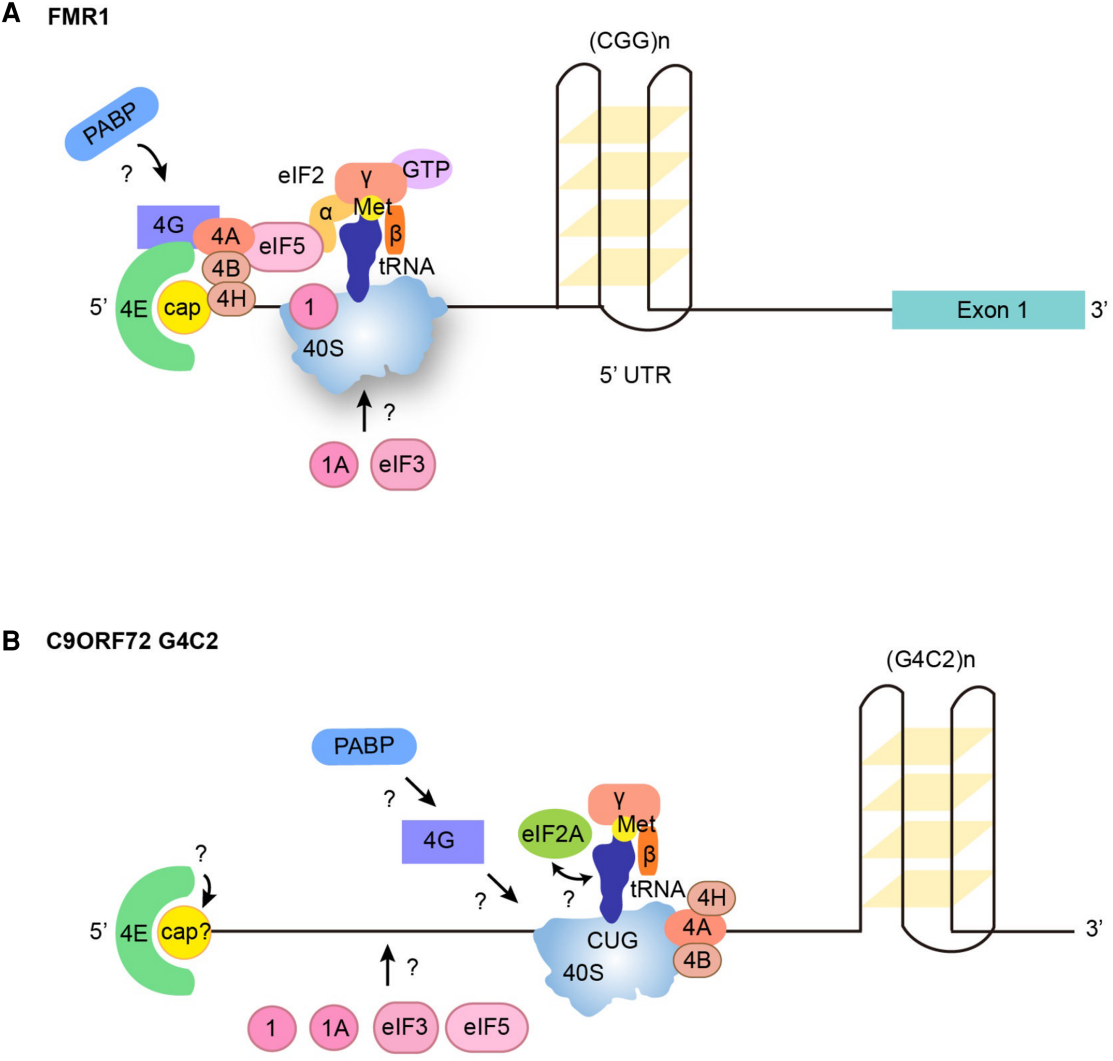
Known RNA structures and protein factors involved in RAN translation. (**A**) RAN translation of *FMR1* occurs in a Cap-, eIF4E- and eIF4A-dependent manner along with eIF4A cofactors eIF4H and eIF4B, recruiting the 40S ribosome and eIF2α-bound Met-tRNA_i_^Met^ to the near-cognate ACG start codon upstream of the CGG repeat expansion. Regulation of start codon fidelity through eIF1 and eIF5 is important. Any potential role of the translation initiation factors PABP, eIF4G, eIF1A and eIF3 remain unknown. (**B**) RAN translation of the GGGGCC repeat expansion from *C9ORF72* occurs in an eIF4A-dependent manner to recruit the 40S ribosome subunit and eIF2A-bound Met-tRNA_i_^Met^ to the near cognate CUG start codon upstream of the hexanucleotide repeat expansion. The eIF4A cofactors eIF4B and eIF4H have been shown to be disease modifiers and are involved. Contradictory evidence for the role of the m^7^cap and eIF4E factor indicates that further elucidation of their roles is required. Any potential role of the translation initiation factors PABP, eIF4G, eIF1, eIF1A, eIF3 and eIF5 still remain unknown. The mechanisms involved in the RAN translation of *C9ORF72* antisense CCCCGG-repeat transcripts have not yet been explored.

An improved understanding of the mechanisms driving RAN translation has begun to emerge with a clear role for the general translation initiation factor eIF4A, a DEAD-box RNA helicase, identified in stimulating the canonical translation of mRNAs containing complex secondary structures such as G-quadruplexes in the 5′-UTR of oncogenes [85]. The identification of inhibitors of eIF4A highlighted that the RNA helicase activity of eIF4A plays an essential role in unwinding secondary structures during ribosome scanning [86–88]. G-quadruplex structures are formed in GGGGCC-repeat [89] and CGG-repeat [90] RNAs. The eIF4A inhibitor hippuristanol showed that eIF4A is required for the RAN translation of CGG-repeat expansions in *FMR1* in FXTAS [47] (Figure 2A) and GGGGCC-repeat transcripts in C9ORF72-ALS/FTD [91] (Figure 2B). The eIF4A inhibitor FL3 [92] further confirmed the role of eIF4A in the RAN translation of sense *C9ORF72*-repeat transcripts. However, the RAN translation mechanisms of antisense *C9ORF72*-repeats which form a double RNA helix [93] remain completely unknown.

The RNA helicase activity of eIF4A is significantly enhanced by two cofactors, eIF4B and eIF4H [94–96], and eIF4B is essential for the translation of mRNAs with long-structured 5-UTRs independently of eIF4A [97]. Interestingly, recent *Drosophila* screens involving sense *C9ORF72*-repeat [98] and FXTAS CGG-repeat [99] transcripts identified eIF4B and eIF4H as disease modifiers of the RAN translation with down-regulation of eIF4B or eIF4H leading to reduced RAN translation and associated toxicity, ameliorating *Drosophila* eye neurodegenerative phenotypes and life span (Figure 2A,B). Interestingly, sequestration of eIF4H by GGGGCC-repeat sequences was previously reported [43]. DDX3X, another RNA helicase which is required for the resolution of RNA–RNA structures in long GC-rich 5′-UTRs, is also implicated in the RAN translation of FXTAS CGG-repeats, with suppression of this helicase inhibiting RAN translation and rescuing associated toxicity in *Drosophila* and primary neurons [99]. However, the role of DDX3X in RAN translation appears sequence-specific since the depletion and overexpression of DDX3X respectively lead to increased and reduced DPR levels in C9ORF72-ALS lymphoblasts [100]. Interestingly, the ribosomal protein RPS25, involved in IRES translation [101], also behaves differently during RAN translation of FXTAS CGG-repeats [99] and sense *C9ORF72*-repeats [102]. Suppression of RPS25 in a FXTAS *Drosophila* model enhanced RAN-translated protein production and associated toxicity [99], while suppression of RPS25 reduced DPR production and rescued associated toxicity in yeast, *Drosophila* and human C9ORF72-ALS/FTD models [102].

RAN translation of reporter constructs require both a m7G-cap and eIF4E for FXTAS CGG-repeats [47] and sense *C9ORF72*-repeats [91]. Accordingly, the eIF4E competitive inhibitor m7G-cap analogue (m7GpppG) prevented RAN translation of FXTAS CGG-repeats [47] and sense C9ORF72-repeats [91,92]. However, another study using a bicistronic reporter construct with all-frame stop codons prior to the initiating start codon reported that RAN translation of *C9ORF72-*repeat transcripts still occurred, suggesting recruitment of ribosomes in a cap-independent manner [103]. eIF4E was shown to be important for the RAN translation of sense *C9ORF72*-repeat transcripts using the 4EIRCat inhibitor [92] however, it was also reported that depletion of eIF4E does not result in a reduction in RAN translation [103] (Figure 2A,B). It thus clearly appears that the sequence-specific context surrounding repeat expansions regulate the mechanisms of RAN translation.

Additional translation initiation factors involved in start codon fidelity are implicated in RAN translation. eIF1 is important in increasing AUG start codon fidelity and overexpression reduces RAN translation and associated toxicity in FXTAS *Drosophila* [99]. eIF5, on the other hand, relaxes start codon fidelity and suppression of eIF5 reduces RAN translation and associated toxicity in FXTAS *Drosophila* [99] (Figure 2A). SCA8 has been shown to require eIF3f during RAN translation of poly-serine and poly-alanine proteins [104]. eIF3f is a non-core component of eIF3 which plays a role in regulating both canonical and IRES-dependent translation [105]. Interestingly, the poly-S proteins produced by RAN translation in SCA8 [104] and HD [52] and poly-QAGR/LPAC in DM2 [106] accumulate in white matter brain regions, where eIF3f levels are elevated compared with grey matter [107], further supporting a potential role of eIF3f in the RAN translation and/or its regulation for some repeat expansions.

### The roles of near-cognate and non-cognate initiation codons in non-AUG translation initiation

Complex secondary RNA-repeat structures such as G-quadruplexes appear to play direct initiating roles through altered ribosome scanning and/or recruitment of ribosomes at near-cognate initiation codons, which differ from AUG by only one nucleotide, and non-cognate initiation codons that differ by more than one nucleotide. FXTAS CGG-repeats within the 5′-UTR of *FMR1* initiate RAN translation of FMRpolyG at a near-cognate ACG codon embedded in a putative Kozak element 32 nucleotides upstream of the repeats in the +1 reading frame, while FMRpolyA initiates at a non-cognate GCG codon within the repeats in a +2 reading frame [47,48]. Mass spectrometry analysis also indicated that polyA RAN-translated proteins from the SCA8 CAG repeats are initiated at non-cognate GCA codons throughout the repeat tract [45] while non-cognate CUU and ACU codons are used to initiate RAN translation of polyQ upstream of the SCA3 CAG repeats and polyA proteins likely within the repeats [108] in transfected cell models. Sense *C9ORF72*-repeat transcripts initiate RAN translation with a Met-tRNAi^Met^ through eIF2A at a Kozak-embedded CUG codon located 24 nucleotides upstream of the repeat sequence in the +1 reading frame of transfected reporter constructs [91,92,109].

### The integrated stress response enhances non-canonical translation

**Figure 3.**
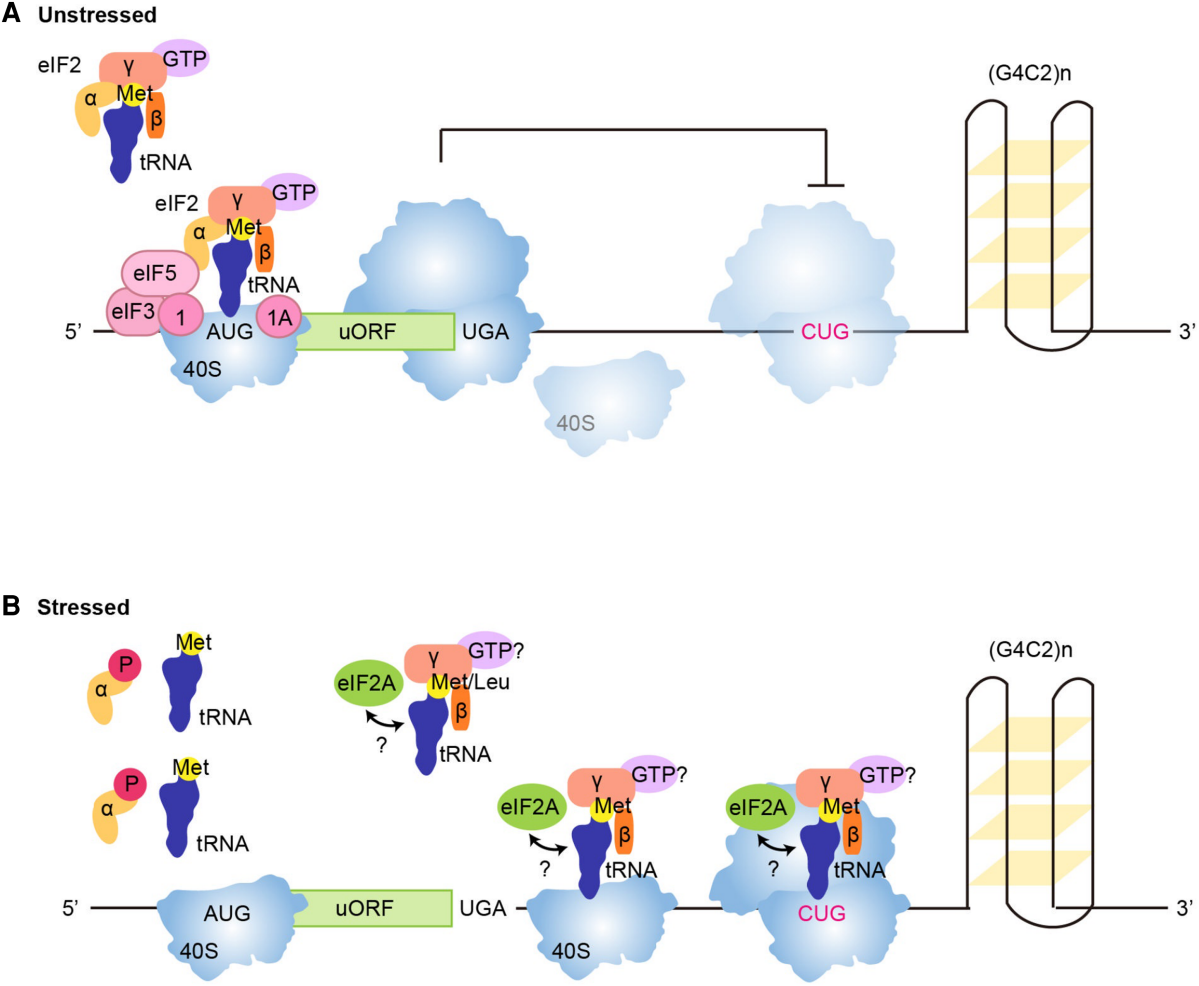
The role of a uORF in the translation of RAN proteins from pathological sense GGGGCC *C9ORF72* repeat expansions. (**A**) In unstressed cellular conditions, a uORF of 55 nucleotides in length within intron 1 of *C9ORF72* inhibits RAN translation of the downstream repeat expansion. The uORF is translated through the canonical translation machinery and ribosomes are unable to reassemble on the mRNA for initiation at the downstream CUG RAN initiation codon. (**B**) Following cellular stress, phosphorylation of eIF2α prevents its binding of Met-tRNA_i_^Met^ results in an inhibition of eIF2-driven canonical translation. Consequently, the alternative tRNA recruiting factor eIF2A is able to initiate non-canonical translation. The scanning 40S ribosomal complex scans through the uORF and eIF2A initiates RAN translation at the downstream CUG codon.

Under non-stressed conditions, RAN translation of sense C9ORF72-repeats is strongly inhibited by an upstream open reading frame (uORF) of 55 nucleotides which is located in intron 1 and flanked by an AUG start codon and 2 downstream stop codons (UGA and UAA) [92] (Figure 3A). However, the integrated stress response (ISR), which is stimulated during disease progression, leads to phosphorylation of eIF2α and down-regulation of canonical translation due to poor recruitment of the 60S subunit of the ribosome and inhibition of translation initiation. This results in read-through of the uORF and subsequent initiation at the downstream CUG codon responsible for RAN translation of the hexanucleotide-repeats [92] (Figure 3B). In neurons, eIF2α phosphorylation is important for synaptic plasticity and rapid activity-dependent alterations of synaptic proteins [110,111]. Following cellular stress and activation of the ISR, a shift in translation occurs towards a subset of transcripts with 5′-UTRs containing uORFs, e.g. ATF4 and CHOP [112], cellular IRES e.g. HIAP2, HIF1α and VEGF [113] and non-AUG start codons e.g. EPRS and major histocompatibility class I antigens [81,114], allowing these transcripts to escape eIF2α-phosphorylated translational inhibition. Phosphorylation of eIF2α increases non-canonical translation and increasing ISR occur concomitantly with increased RAN translation of both FMR1 and C9ORF72 repeats in a positive feedback loop [91,103,109,115]. It is noteworthy that ISR-resistant translation involves eIF2D, eIF2A and eIF5B [116–119] and as aforementioned, RAN translation of sense *C9ORF72*-repeats initiates at a CUG codon through eIF2A [109] (Figure 3B). Taken together, these studies strongly support a model in which increased ISR upon disease progression down-regulates canonical translation of the uORF to stimulate RAN translation in a positive disease-enhancing feedback loop.

## Conclusions

The pathogenesis driven by nucleic acid repeat expansions and RAN-translated products is complex and still poorly understood. Multiple mechanisms of neuronal injury involve toxic RNA gain-of-functions, haploinsufficiency as well as protein gain-of-functions via canonical translation of proteins with extended polyQ domains and/or RAN translation of toxic repeat polypeptides which have been characterised *in vitro* in 13 reporter repeat expansion cell models and in patient bio-samples from seven diseases, including SCA8, DM1 [45,106], C9ORF72-ALS [49–51,120] and HD [52]. However, the molecular mechanisms involved in RAN translation remain poorly understood, hindering thus the development of therapeutic approaches for this incurable group of diseases. In addition, it has remained challenging to dissect how the life-long expression of repeat transcripts and RAN-translated proteins contribute to the pathogenesis of these progressive adult-onset diseases. In the past few years, growing evidence has implicated RAN translation as one of the main drivers of neurotoxicity in C9ORF72-ALS/FTD models. On the other hand, FXTAS was initially thought to be caused by intranuclear retention of transcripts and sequestration of splicing factors [40,41] however, the later discovery of RAN translation in the same model challenged this view [46]. Similarly in C9ORF72-ALS, increasing the number of intranuclear RNA foci does not appear to alter neuronal survival or global RNA processing while expression of DPRs drives the neurodegeneration process [44,54,121]. Sequestration of RNA-binding factors on repeat transcripts might not only necessarily implicate loss-of-function mechanisms, as often hypothesised, as cells can use compensatory mechanisms to up-regulate the transcription/translation of the sequestered proteins. So far, there has not been any evidence demonstrating that RNA foci and sequestration of proteins on RNA-repeats trigger protein loss-of-function mechanisms. In contrast, RNA-repeat sequestration of SRSF1 produces protein gain-of-function by driving the nuclear export of intron-retaining C9ORF72-repeat pre-mRNAs and subsequent RAN translation of cytotoxic DPRs in the cytoplasm [44].

If expression of polymeric repeat proteins can kill cells and animal models, it is difficult to evaluate which levels are RAN-translated in patients and which expression levels trigger toxicity depending on the nature of the repeat expansion, disease and cell type. Understanding the molecular mechanisms of RAN translation will be key to improving our understanding of pathogenesis. Additional mechanisms such as ribosome frameshifting events also need to be explored. For example, frameshifting was suggested to occur during translation of CAG-repeat expanded transcripts in the −1 frame in SCA3 [122–124] as well as into the −1 [125] and +1 [126] directions in HD, however, the production of chimeric repeat proteins was not directly evidenced. RAN translation of three C9ORF72-related DPRs encoded from the three reading frames of sense repeat transcripts suggested that RAN translation initiates at multiple initiation sites [91,109], however, mutation of a near-cognate CUG start codon also inhibited the production of all sense DPRs, suggesting the occurrence of potential frameshifting mechanisms that remain to be demonstrated [92].

## Perspectives

- Mechanisms of RAN translation and RAN-translated proteins/peptides still remain poorly characterised despite discovery in DM1/SCA8 patient samples in 2011, and later in C9ORF72-ALS and HD in 2013 and 2015, respectively.
- RAN-translation occurs in absence of the canonical methionine start codon, in all frames, and from coding and non-coding regions of transcripts encoding proteins of various functions. So far, it is known to involve RNA secondary structures formed by repeat sequences and general translation initiation factors, which exhibit/stimulate RNA-helicase activities, or play a role in the fidelity of start codon recognition.
- In the future, it will be fundamental to fully identify the RAN-translation machinery components and mechanisms in the context of the sequences flanking each microsatellite repeat expansions, as well as examine further the pathological contribution of RAN-translated products for the future development of much-needed disease treatments.

## Open Access Statement

Open access for this article was enabled by the participation of University of Sheffield in an all-inclusive Read & Publish pilot with Portland Press and the Biochemical Society under a transformative agreement with JISC.

## Abbreviations

ALS: amyotrophic lateral sclerosis
C9ORF72: chromosome 9 open reading frame 72
DM1: dystrophy type 1
DPRs: dipeptide-repeat proteins
FMR1: fragile X mental retardation 1
FTD: frontotemporal dementia
FXS: Fragile X syndrome
FXTAS: fragile X-associated tremor ataxia syndrome
HD: Huntington's disease
IRES: internal ribosome entry site
ISR: integrated stress response
RAN: repeat-associated non-AUG
SCA8: spinocerebellar ataxia type 8
UTR: untranslated regions
